# The Plasmid Complement of the Cheese Isolate *Lactococcus garvieae* IPLA 31405 Revealed Adaptation to the Dairy Environment

**DOI:** 10.1371/journal.pone.0126101

**Published:** 2015-05-05

**Authors:** Ana Belén Flórez, Baltasar Mayo

**Affiliations:** Departamento de Microbiología y Bioquímica, Instituto de Productos Lácteos de Asturias (IPLA-CSIC), Carretera de Infiesto, s/n, 33300-Villaviciosa, Asturias, Spain; Université Laval, CANADA

## Abstract

*Lactococcus garvieae* is a lactic acid bacterium found in raw-milk dairy products as well as a range of aquatic and terrestrial environments. The plasmids in *L*. *garvieae* have received little attention compared to those of dairy *Lactococcus lactis*, in which the genes carried by these extrachromosomal elements are considered of adaptive value. The present work reports the sequencing and analysis of the plasmid complement of *L*. *garvieae* IPLA 31405, a strain isolated from a traditional, Spanish, starter-free cheese made from raw-milk. It consists of pLG9 and pLG42, of 9,124 and 42,240 nucleotides, respectively. Based on sequence and structural homology in the putative origin of replication (*ori*) region, pLG9 and pLG42 are predicted to replicate via a theta mechanism. Real-time, quantitative PCR showed the number of copies per chromosome equivalent of pLG9 and pLG42 to be around two and five, respectively. Sequence analysis identified eight complete open reading frames (*orf*s) in pLG9 and 36 in pLG42; these were organized into functional modules or cassettes containing different numbers of genes. These modules were flanked by complete or interrupted insertion sequence (IS)-like elements. Among the modules of pLG42 was a gene cluster encoding specific components of a phosphoenolpyruvate-phosphotransferase (PEP-PTS) system, including a phospho-β-galacosidase. The cluster showed a complete nucleotide identity respect to that in plasmids of *L*. *lactis*. Loss of pLG42 showed this to be involved in lactose assimilation. In the same plasmid, an operon encoding a type I restriction/modification (R/M) system was also identified. The specificity of this R/M system might be broadened by different R/M specificity subunits detected in pLG9 and in the bacterial chromosome. However, challenges of *L*. *garvieae* IPLA 31405 against *L*. *lactis* phages proved that the R/M system was not involved in phage resistance. Together, these results support the hypothesis that, as in *L*. *lactis*, pLG42 contribute towards the adaptation of *L*. *garvieae* to the dairy environment.

## Introduction

*Lactococcus garvieae* is a lactic acid bacterium (LAB) that was first isolated from the udder of a cow with mastitis [1]. It is now documented as an animal pathogen, causing mastitis in ruminants [2] and lactococcosis in marine and freshwater fish [3]. Human *L*. *garvieae* infections are rare, but cases of opportunistic endocarditis and spondylodiscitis have also been reported [4]. Further, this microorganism is found in many farmhouse dairy products manufactured from raw milk, from which it is occasionally retrieved as a majority component of their native microbiota [5–8]. Its widespread distribution suggests the species can adapt to many environments.

Dairy and environmental strains of *Lactococcus lactis* can carry a complex extrachromosomal complement consisting of many plasmids [9, 10]; these are thought to provide genes of use in adaptation to new environments [11–13]. Certainly, the plasmids of *L*. *lactis* mediate extensive horizontal gene transfer (HGT) and rearrangements, enabling the species to acquire and recruit traits that confer selective advantages in terms of colonizing and persisting in different niches [9, 14]. Starter *L*. *lactis* strains possess plasmids that confer upon them properties important for growing in milk, such as extracellular caseinolytic activity, the ability to rapidly utilise lactose via a phosphoenolpyruvate-phosphotransferase system (PEP-PTS), the ability to assimilate citrate, different phage resistance mechanisms, production of exopolysaccharides and bacteriocins, etc. [11, 12, 15]. These properties are pivotal in the use of these cultures as starters in industrial fermentations. In contrast, *L*. *lactis* strains from non-dairy (plant) sources contain plasmids with genes that allow complex polysaccharides to be utilised and different metals to be taken up [13, 16]; these properties are unimportant for bacteria growing in milk.

The plasmids carried by *L*. *garvieae* received little attention until quite recently. A conjugative plasmid conferring multidrug resistance, pKL0018, has been identified in a pathogenic *L*. *garvieae* strain isolated from a yellowtail fish [17]. pKL0018 contains two *ermB* genes and one *tetS* gene in an *Enterococcus faecalis*-related plasmid backbone. Even more recently, genome analysis of an *L*. *garvieae* strain of clinical origin allowed its whole plasmid complement to be characterised [18]. It was found to consist of five plasmids (pLG1 to pLG5), in which genes coding for virulence and pathogenic factors were identified. This strongly suggests that, as in other bacteria, the plasmids of *L*. *garvieae* serve adaptive purposes. The study of *L*. *garvieae* plasmids may therefore help us understand the importance of these elements in the adaptation of the species to the different ecological niches it occupies. Additionally, the plasmids of *L*. *garvieae* might be of use as future biotechnological tools; the lack of species-specific cloning vectors and techniques have to date hindered molecular studies being undertaken [19].

The present work reports the sequencing and analysis of the plasmid complement of *L*. *garvieae* IPLA 31405, a dominant strain isolated from a traditional cheese made from raw milk without added starters [8]. The genome sequence of IPLA 31405 has already been reported [20]. Genome analysis, PCR amplification, sequencing and hybridisation techniques were used to obtain, analyse, annotate and characterize the sequences of the two plasmids identified in this bacterium.

## Material and Methods

### Bacterial strains and growth conditions

*Lactococcus garvieae* IPLA 31405 was isolated from among the dominant microbiota of a traditional raw-milk cheese [8]. In some assays, *L*. *garvieae* CECT 4531^T^ (from the Spanish Type Culture Collection), N201, 1042, and 1204 from Salers raw milk cheese [21], and DK2-25 from a Serbian traditional fermented milk [22], were also used as controls. *Lactococcus lactis* subsp. *cremoris* MG 1614, a plasmid-free, lactose negative strain resistant to streptomycin (500 μg ml^-1^) was used as a recipient in conjugation experiments. Otherwise stated, bacteria were grown in static under aerobic conditions in M17 broth or agar (Oxoid, Basingstoke Hampshire, UK) supplemented with 1% glucose (GM17) or lactose (LM17) at 30°C for 24 h.

### DNA isolation

The isolation of plasmid DNA was performed essentially according to the method of O’sullivan and Klaenhammer [23]. Instead of using the original solutions, the denaturation and neutralization steps were performed using the solutions provided with the commercial Plasmid Mini Kit (Qiagen, Hilden, Germany). Plasmid profiles were prepared by electrophoresis in 0.75% agarose gels in 1 x TAE buffer (40 mM Tris, 20 mM acetic acid, and 1 mM EDTA), stained with ethidium bromide (0.5 mg mL^-1^), and visualized and photographed under UV light.

Total genomic DNA was purified using the ATP Genomic Mini Kit (ATP Biotech, Taipei, Taiwan) following the manufacturer’s recommendations.

### DNA sequencing and bioinformatics analysis

Putative plasmid sequences of pLG9 and pLG42 from *L*. *garvieae* IPLA 31405 were retrieved from the published whole genome sequence data [20]. Primers based on the sequences at the beginning and the end of the contigs containing plasmid sequences were designed and used in PCR reactions for gap closing and/or sequence verification, employing plasmid DNA from IPLA 31405 as a template. Amplifications were performed in a reaction mixture of 50 μl containing 2 μl of purified DNA (50 ng), 25 μl of 2 x Taq master Mix (Ampliqon, Odense, Denmark), 1.5 μl of each primer (10 μM) and 20 μl H_2_O. The PCR conditions were as follow: an initial denaturation cycle at 95°C for 5 min, 35 cycles of a denaturation step at 94°C for 30 s, an annealing step at 50°C for 1 min, an extension step at 72°C for 2 min, and a final extension cycle at 72°C for 10 min. PCR amplicons were examined in 1% agarose gels and stained and photographed as above. Finally, amplicons were purified using the ATP Gel/PCR Extraction Kit (ATP Biotech) and sequenced by cycle extension in an ABI 373 DNA sequencer (Applied Biosystems; Thermo Scientific, Waltham, MA, USA).

Plasmids sequences were assembled using the Vector NTI computer program (Invitrogen; Thermo Scientific). This program was also used to search the DNA sequences for putative open reading frames (*orf*s). Predicted *orf*s were then manually inspected for homology against the NCBI non-redundant DNA and protein databases using the online BLAST programme (http://blast.ncbi.nlm.nih.gov/Blast.cgi). *orf*s whose DNA sequences overlapped with another by more than 20 coded amino acids, and *orf*s shorter than 50 (for pLG9) or 100 (for pLG42) coded amino acids, were not taken into account.

The distance matrix of a multiple alignment between RepB of both pLG9 and pLG42 and those from homologous proteins of representative lactococcal plasmids was used to set up a phylogenetic tree with the neighbor-joining method and a bootstrapping trial number of 1000.

### DNA hybridisation

Total and plasmid DNA was digested with restriction enzymes (Takara, St Germain en Laye, France) and after electrophoresis blotted onto Hybond-N nylon membranes (GE Healthcare Bio-Sciences, Buckinghamshire, UK) using a standard protocol [24]. Internal segments of the 6-phospho-β-galactosidase gene (*lacG*) and the gene encoding the replication protein of pLG9 (*repB*), both amplified by PCR (Table 1), were used as probes in hybridisation experiments. Labelling with digoxigenin, hybridisation under high-stringency conditions, and detection were performed using the DIG-High Prime DNA Labelling and Detection Starter Kit II (Roche, Basel, Switzerland) following the manufacturer’s recommendations.

**Table 1:** Primers utilized in this study for conventional and real-time PCR amplification.

Primers	Sequence 5’-3’	Annealing temperature (°C)	Amplicon size (bp)	Efficiency
**PCR primers**				
BPG-F	CAGTCTGTGCGTGGAACATA	55	987	-
BPG-R	CTGCTTATCAAGCAGAAGGT			
RepB-pLG9-F	CAAGTGCTCTTTGACACCAT	55	1007	-
RepB-pLG9-R	CAGGTGCTGACCTTGAATGA			
**Real-time PCR (qPCR) primers**				
qPCR-EF-Tu-F	TTGAGGTTCACCGTTCAAAGC	60	70	0.9793
qPCR-EF-Tu-R	CGACTTCCCAGGTGACGATAC			
qPCR-GADPH-F	CGACCTTACAGATCCAGCAATG	60	68	0.9847
qPCR-GADPH-R	CGTCGAAACGACCTTGAGTTG			
qPCR-repB-pLG9-F	AACCAATACGAGCATTACAGTGTCA	60	66	0.9724
qPCR-repB-pLG9-R	ATTGCGGTATGCTTCCACTTG			
qPCR-repB-pLG42-F	ATACGAGGCTATTGTTGGAACATTT	60	76	0.9965
qPCR-repB-pLG42-R	TGTAACCCTACCATGATTGATCGA			

### Determination of the relative plasmid copy number

The relative copy number of plasmids pLG9 and pLG42 was evaluated by quantitative real-time PCR (qPCR) using Power SYBER Green PCR Master Mix (Applied Biosystems) and a Fast Real-Time PCR system (Applied Biosystems). *L*. *garvieae* IPLA 31405 was cultured in GM17 at 32°C and DNA extraction performed as before at the beginning and in the middle of the exponential growth phase, and again during the stationary phase. PCR primers were designed for genes coding for replication proteins (*repB*-pLG9 and *repB*-pLG42) (Table 1) using Primer Express software (Applied Biosystems). The chromosomally-encoded, single-copy genes coding for glyceraldehyde-3-phosphate-dehydrogenase (GADPH) and elongation factor Tu (EF-Tu) were used as reporter control genes. Primers for the control genes (Table 1) were based on the published genome sequence of IPLA 31405 [20]. The relative copy number was calculated using the formula *N*_*relative*_ *= (1+E)* ^*-ΔCt*^ [25], where *E* is the amplification efficiency and *ΔCt* the difference between the threshold cycle number (*Ct*) of target and reference genes. The experiments were performed in triplicate; mean values are provided.

### Plasmid stability

The stability of the plasmids was assayed after growing the cells in non-selective GM17 medium for approximately 100 generations (5 days). Ten-fold dilutions were daily plated onto GM17 agar plates and incubated at 30°C for 24 h. Fifty colonies were then picked at random and used directly in independent PCR amplifications using specific primer pairs for pLG9 (targeting the repB gene) and pLG42 (targeting the phospho-β-galactosidase gene) (Table 1).

### Mating procedure

Recipient and donor strains were grown separately on GM17 or LM17, respectively, at 30°C for 16–18 h. After incubation, they were mixed at a donor:recipient ratio of 10:1. Aliquots of the mating mixtures were filtered through 0.45 μm nitrocellulose filters, which were then incubated over the surface of GM17 agar plates. Matings were allowed to proceed for 24 h at 30°C, after which the filters were suspended in fresh M17 broth without sugar. Serial dilutions of this medium were plated on GM17 with streptomycin (500 μg mL^-1^) or on LM17 for separate counting of recipient and donor strains, respectively. Transconjugants were selected on LM17 agar plates with streptomycin.

### Phenotypic analysis

Strains that lost their plasmid(s) were selected for phenotype analysis in order to further verify connections between gene content of plasmids and phenotypic properties.

#### Sugar fermentation

Parental and pLG-free derivative strains were tested for their carbohydrate fermentation ability using the API-CHL system as recommended by the supplier (bioMérieux, Marcy l’Etoile, France).

#### Growth in lactose and in milk

Parental and plasmid-free strains were grown in M17 broth containing glucose and lactose. Maximum growth rate (h^-1^) was determined as follows: μ_max_ = (ln x_1_—ln x_0_)/(t_1_—t_0_). The acidification ability of the strains was assayed in UHT-treated milk (CAPSA, Siero, Spain) with and without added yeast extract (0.5%).

#### Heavy-metal resistance

The minimum inhibitory concentration (MIC) of a series of heavy metals was determined in *L*. *garvieae* IPLA 31405 and its plasmid-free derivatives by inoculating the strains into LSM (90% IsoSensitest broth and 10% MRS broth; both from Oxoid; Thermo Scientific) [26](Klare et al., 2005). Two-fold increasing concentrations (from 0.03 to 2048 μg/ml) of the following metal salts were assayed: cadmium (CdSO_4_·8H_2_O), cobalt (CoCl_2_·6H_2_O), copper (CuSO_4_·5H_2_O), iron (FeC_6_H_5_O_7_·5H_2_O and FeSO_4_·7H_2_O), lead (Pb(NO_3_)_2_), magnesium (MgSO4·7H_2_O), manganese (MnSO4·H_2_O), mercury (HgCl_2_) and zinc (ZnSO4·7H_2_O). A bacterial suspension corresponding to McFarland standard 1 in sodium chloride (0.9%) was prepared and diluted 1:1000. This was then used to inoculate metal-containing LSM broth to obtain a final cell concentration of ~10^5^ cfu/ml. Readings were recorded after 24 h of incubation at 30°C; the MICs were taken as the lowest concentration at which growth was completely inhibited.

#### Phage resistance

Rapid screening of *L*. *garvieae* strains for phage resistance/susceptibility was performed by using an agar spot test technique. Briefly, GMI7 agar plates were overlaid with top lawns of soft GM17 agar (0.7%, wt/vol) containing 100 μL of an overnight culture of each strain, 5 mM CaCl2, and 0.75% (wt/vol) glycine. Cell lawns were spotted with 20 μL of purified phages or phage lysates at estimated titres between 10^8^ to 10^10^ plaque forming units (pfu) per mL, incubated overnight at 30°C, and then examined for the presence of halos of clearance.

### GenBank accession number

The nucleotide sequences of pLG9 and pLG42 were deposited in the GenBank database under accession numbers KM007159 and KM007160, respectively.

## Results and Discussion

### General plasmid features

**Fig 1:**
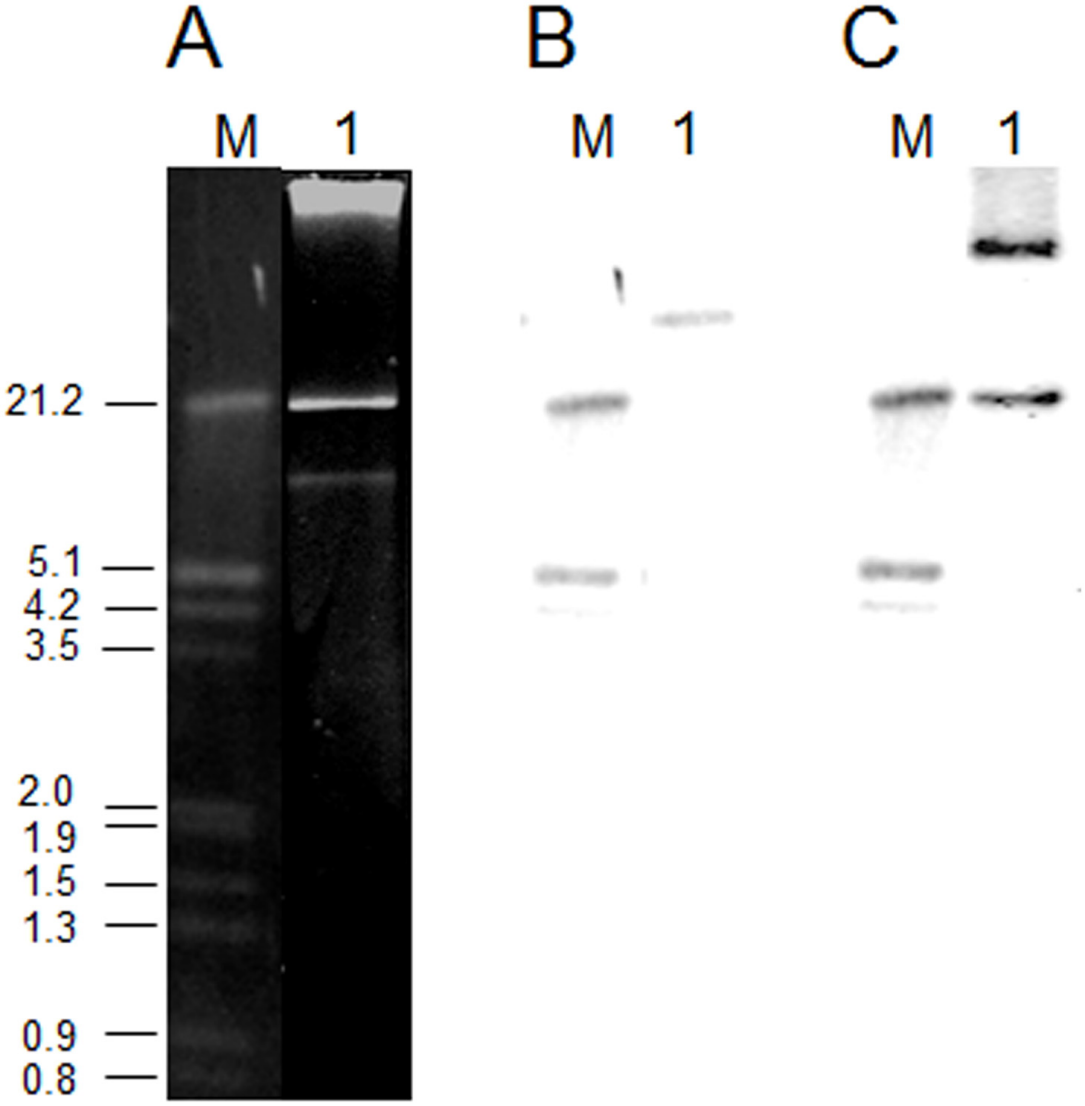
**Panel A.** Agarose gel electrophoretogram of the plasmid profile of *Lactococcus garvieae* 31405 (line 1). M, molecular weight marker (lambda DNA digested with EcoRI and HindIII). **Panel B.** Autoradiogram of the gel in panel A hybridized with a DIG-labelled probe derived by PCR from an internal segment of the *repB* gene of pGL9. **Panel C.** Autoradiogram of the gel in panel A hybridized with a DIG-labelled probe derived by PCR from an internal segment of the phospho-β-galactosidase gene.

*L*. *garvieae* IPLA 31405 was found to contain two plasmid bands (Fig 1A). Digestion of the profile with single and double combinations of the restriction enzymes XhoI, BamHI, StuI, NheI and Pst estimated the size of the plasmids to be of approximately 40 and 10 kbp (not shown). The draft genome sequence of IPLA 31405 included 23 contigs from 598 to 1,017,382 bp [20]. DNA and deduced protein sequences from the contigs were individually subjected to BLASTN and BLASTP analysis (http://blast.ncbi.nlm.nih.gov). Two *orf*s encoding plasmid-replication proteins were identified, suggesting that the two bands in the plasmid profile belonged to different molecules. For filling in the gaps and ordering the contigs, contig sequences considered to be plasmid-related were used to design oligonucleotide primers, which were then used in PCR amplifications using plasmid DNA from IPLA 31405 as a template. The amplicons obtained were sequenced, and the new sequences assembled with the existing ones. Analysis of the closed, circular sequences resulted in two plasmids, pLG9 and pLG42, of 9124 and 42,459 bp, respectively. The G+C content of pLG9 was 32.6%, and for LG42 it was 35.3%, slightly lower than that of the *L*. *garvieae* IPLA 31405 genome sequence (38.5%) [20]. In terms of the total genome (chromosome and plasmids), the plasmid complement represents less than 2.5% of the total genomic DNA. Hybridisation experiments using internal segments of one of the two replicating genes and a phospho-β-galactosidase gene as probes (Fig 1B and 1C) proved unequivocally that the plasmid complement of IPLA 31405 involved two plasmids.

At the nucleotide level, a 1.5 kbp segment of pLG9, carrying the putative origin of replication (*ori*) region and the replication protein (*repB*) gene, showed the greatest similarity to the corresponding *ori* region of pL2 (DQ917780.1) from *L*. *lactis* subsp. *lactis* [27], and was very similar to that of other plasmids from *L*. *lactis* (pVS40, L02920.1; pNZ4000, NC_002137.1; pCV56D, CP002369.1; etc.) and *L*. *garvieae* (pLG3) (NC_016970.1). As for pLG42, the nucleotide sequence of an 8.7 kbp fragment proved to be almost identical to the lactose utilization region of pVF50 (JN225497.1) [13], and its equivalent gene cluster in many lactose plasmids from *L*. *lactis*. Moreover, the plasmid segment encoding a Type I restriction-modification system shared complete nucleotide identity with those encoded by plasmids pAH82 (NC_004966.1) [28] and pVF21 (JN172911.1) [13] from *L*. *lactis*.

**Fig 2:**
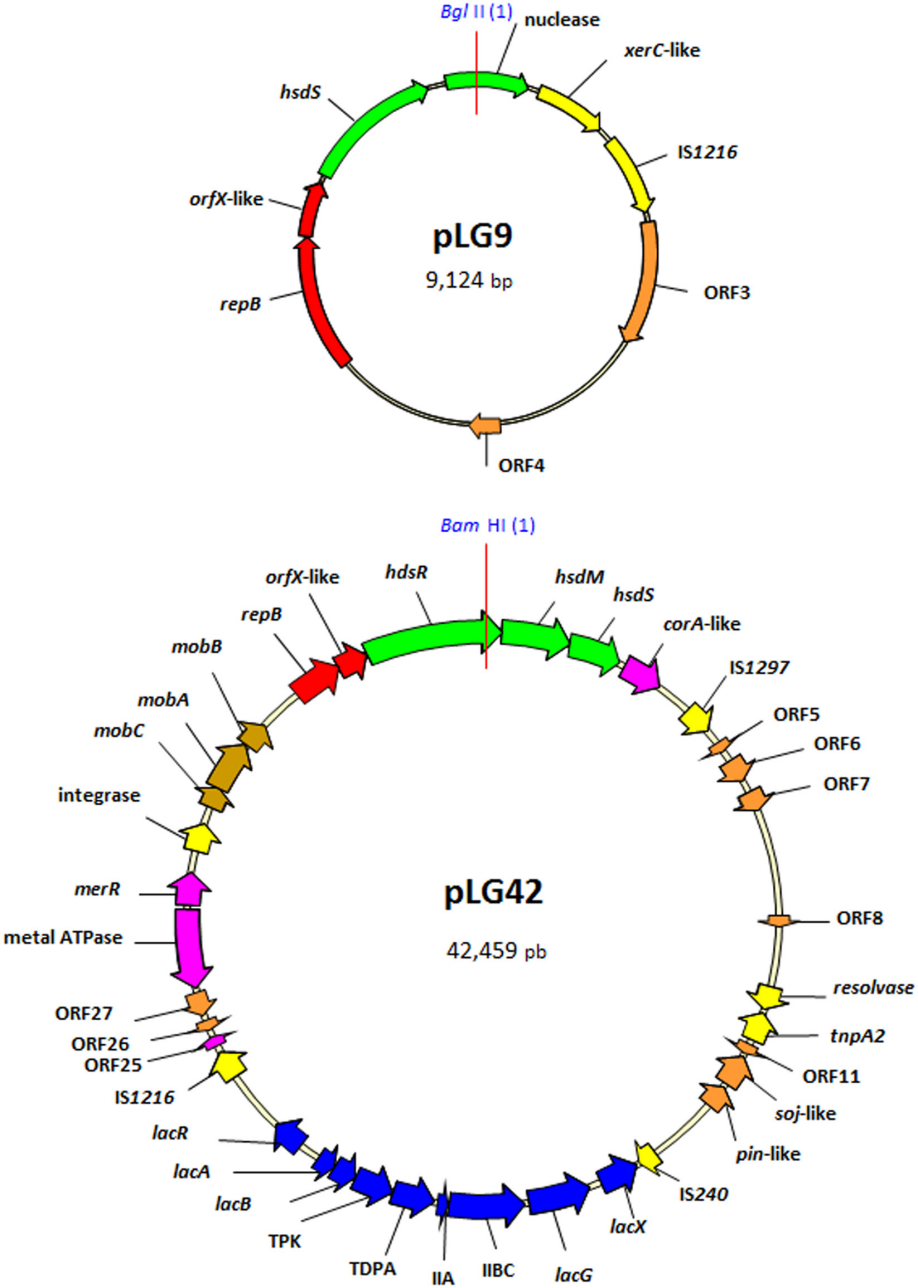
Genetic organization of pLG9 and pLG42 plasmids, including position of relevant restriction enzymes and direction and approximate length of genes and open reading frames (*orf*s). Key of colors: in red, genes involved in replication; in yellow, *orf*s of insertion sequences and integrase-related genes; in green, component genes of type I restriction modification systems; in purple, genes involved in the transport of heavy metals (Cd, Hg, Pb); in brown, *orf*s of a plasmid mobilization system; in blue, (*orf*13-*orf*20) genes involved in lactose utilization, including a gene encoding a beta-fosfogalactosidasa (*orf*14) and the regulator (*orf*21); in orange, *orf*s for other genes.

Both pLG9 and pLG42 seemed to be organized into functional modules or cassettes encompassing variable numbers of *orf*s (Fig 2). This was more obvious in the case of pLG42, in which five to six modules were noted. Sequence analysis suggested that these modules, the majority of which have been described in *L*. *lactis*, are of different origin. Except for the replication region of pLG9, none of the modules of either pLG42 or pLG9 showed significant structural nor functional homology to those of pathogenic *L*. *garvieae* strains from fish [17] or humans [18]. In total, eight complete and three disrupted *orf*s were seen to be encoded by pLG9, while pLG42 showed 36 complete and six incomplete *orf*s (Table 2). The region encompassing *orf11* to *orf13* on pLG42 might form part of a par locus that secures the equal distribution of plasmid copies to daughter cells at cell division [29]. This system would be responsible for (i) partitioning of the plasmids, and (ii) prevention of the appearance of plasmid-free segregates. The same genes encoding Soj-like and Pin-like proteins are present in many large lactococcal and enterococcal plasmids, including pK214 (NC_009751.1) and pRE25 (X92945.2) respectively. The modules are flanked by complete or truncated insertion sequence (IS)-like elements harbouring genes encoding integrase/transposase-like proteins (yellow *orf*s in Fig 2). Nine IS elements, of which one was truncated, were identified in the two IPLA 31405 plasmids (two in pLG9 and seven in pLG42). Complete and partial ISs might provide the nucleotide homology required to mediate in DNA rearrangements in the plasmids, involving gene gain (integrations) or gene loss (deletions).

**Table 2:** Open reading frames (ORFs) identified in plasmids pLG9 and pLG42 from *Lactococcus garvieae* IPLA 31405.

ORF	5’ end position	3’ end position^a^	% GC content^b^	No. of aa^c^	Known protein with the highest homology (microorganism) (length)	% aa identity (length)	GenBank Accession no.
**Plasmid pGL9**							
*orf1*	547	1,137	37.5	196	Integrase/recombinase plasmid-associated protein, putative XerC protein (*Lactococcus lactis*)	99 (196)	WP_023189870.1
*orf2*	1,277	1,957	36.8	226	Transposase IS*1216* (*Aerococcus viridans*)	99 (226)	WP_016897246.1
*orf3*	2,029	3,090	25.8	352	Efflux transporter, fusaric acid resistance protein-like (pfam13515) (*Vagococcus lutrae*)	58 (352)	WP_023606696.1
*Δorf*	3,789	3,998	42.5	69	ABC-type transporter (*Bacillus mycoides*) (92 aa)	38 (69)	WP_006094374.1
*Δorf*	4,108	4,290	39.3	60	Integrase, C-terminus (*Lactococcus lactis*) (106 aa)	93 (59)	WP_014011563.1
*orf4*	4,462	4,740	32.9	92	Cytochrome B (*Lactococcus garvieae*)	100 (92)	WP_003134148.1
*Δorf*	4,712	4,870	35.9	52	MobC mobilization protein, N-terminus (*Lactococcus garvieae*) (164 aa)	84 (164)	YP_006964743.1
*orf5*	5,838	6,998	35.5	385	Replication protein RepB (*Lactococcus garvieae*)	100 (385)	WP_004256086.1
*orf6*	6,995	7,564	30.8	189	OrfX-like replication-associated protein (*Lactococcus garvieae*)	99 (189)	WP_004256084.1
*orf7*	7,552	8,709	35.8	385	Type I restriction endonuclease subunit HsdS (*Lactococcus garvieae*)	100 (385)	WP_004256082.1
*orf8*	8,832	541	32.6	277	Nuclease GIY-YIG motif (*Lactococcus garvieae*)	99 (277)	WP_017369152.1
**Plasmid pGL42**							
*orf1*	358	1,953	40.2	531	Type I restriction-modification system, modification subunit/HsdM (*Lactococcus lactis*)	99 (531)	YP_001032001
*orf2*	1,946	3,100	33.4	384	Type I restriction and modification system, specificity subunit/HsdS (pIL7 *Lactococcus lactis*)	94 (180)	YP_004761557
*orf3*	3,286	4,236	32.0	316	CorA-like Mg2+ and Co2+ co-transporter (*Lactococcus lactis*)	99 (316)	YP_005869709
*Δorf*	4,616	5,011	28.9	131	GIY-YIG ion-binding catalytic domain protein (*Lactococcus raffinolactis*) (277 aa)	100 (125)	ZP_11239917
*orf4*	5,065	5,745	39.6	226	Transposase, element IS*1297* (*Streptococcus thermophilus*)	97 (226)	YP_006339954
*orf5*	6,365	5,982	34.1	127	Hypothetical, thioredoxin-like protein (pGL5p05 *Lactococcus garvieae*)	86 (124)	YP_005352357
*orf6*	6,596	7,168	37.8	185	Hypothetical protein, no relevant homology	-	-
*orf7*	7,458	7,958	40.6	166	Hypothetical protein, no relevant homology	-	-
*orf8*	10,643	10,990	23.9	115	Hypothetical protein, no relevant homology	-	-
*Δorf*	7,993	10,626	41.7	877	CHW repeat-/cell adhesion domain-containing protease and peptidase (*Lactococcus lactis*) (1019 aa)	36 (580)	YP_006998456
*orf9*	12,172	12,726	37.0	184	Resolvase/integrase (pGdh442_04 *Lactococcus lactis*)	99 (184)	YP_001174712
*orf10*	13,493	12,807	37.6	228	Tnp*A2* transposase (pCV56C *Lactococcus lactis*)	99 (228)	YP_005867383
*orf11*	13,918	13,619	27.3	99	Hypothetical protein (pK214_p03 *Lactococcus lactis*)	99 (99)	YP_001429517
*orf12*	14,688	13,921	28.1	255	Plasmid partitioning Soj-like protein (pK214_p02 *Lactococcus lactis*)	99 (255)	YP_001429516
*orf13*	15,354	14,782	29	190	Site-specific recombinase, DNA invertase Pin-like protein (pK214_01 *Lactococcus lactis*)	97 (190)	YP_001429515
*Δorf*	16,783	16,935	34	51	IS1216, transposase family protein (*Enterococcus faecium*) (177 aa)	100 (51)	WP_002349406
*orf14*	17,004	17,462	41	152	Transposase IS*240* (*Enterococcus faecium*)	97 (152)	ZP_19460855
*orf15*	18,423	17,524	37.5	299	Aldose 1-epimerase, LacX (pLP712 *Lactococcus lactis*)	100 (299)	YP_006965846
*orf16*	20,148	18,715	38.1	477	6-phospho-beta-galactosidase (LacG) (pVF50 *Lactococcus lactis*)	99 (477)	YP_004770083
*orf17*	21,965	20,259	41.0	568	PTS system lactose-specific transporter subunit IIBC (pVF50 *Lactococcus lactis*)	100 (568)	YP_004770084
*orf18*	22,282	21,965	42.1	105	Lactose-specific phosphotransferase enzyme IIA component (*Enterococcus faecium*)	100 (105)	ZP_03980301
*orf19*	23,290	22,310	39.1	326	Tagatose 1,6-diphosphate aldolase (pVF50 *Lactococcus lactis*)	100 (326)	YP_004770086
*orf20*	24,225	23,293	36.9	310	Tagatose-6-phosphate kinase (pVF50 *Lactococcus lactis*)	99 (310)	YP_004770087
*orf21*	24,751	24,236	39.53	171	Galactose-6-phosphate isomerase subunit LacB (pVF50 *Lactococcus lactis*)	99 (171)	YP_004770088
*orf22*	25,193	24,768	38.7	141	Galactose-6-phosphate isomerase subunit LacA (pVF50 *Lactococcus lactis*)	100 (141)	YP_004770089
*orf23*	25,681	26,448	34.9	255	Lactose phosphotransferase system repressor (*Enterococcus faecium*)	99 (255)	ZP_19549772
*orf24*	27,931	28,611	37.6	226	IS*1216* Transposase (pCV56C *Lactococcus lactis*)	99 (228)	YP_005867383
*Δorf*	27,768	27,385	42.5	127	Lead, cadmium, zinc and mercury transporting ATPase, Copper-translocating P-type ATPase (*Lactococcus lactis* subsp. *cremoris*) (394 aa)	98 (127)	YP_005876288
*orf25*	28,797	29,171	31.3	124	Mercuric resistance operon regulatory protein (*Enterococcus faecalis*)	98 (106)	ZP_05475475
*orf26*	29,426	29094	36.2	110	Hypothetical protein, no relevant homology	-	-
*orf27*	30,167	29,505	35.8	220	Ring-infected erythrocyte surface antigen precursor (*Streptococcus macedonicus*)	98 (127)	YP_005094018
*orf28*	32,072	30,168	33.0	643	Lead, cadmium, zinc and mercury transporting ATPase (*Streptococcus macedonicus*)	100 (643)	YP_005094017
*orf29*	32,163	32,942	28.4	259	Putative transcriptional regulator MerR (*Streptococcus macedonicus*)	99 (259)	YP_005094016
*Δorf*	33,444	32995	40.4	149	LcoB (formerly Usp45) copper resistance protein, partial (*Lactococcus garvieae*) (434 aa)	100 (126)	WP_003134158
*orf30*	33,436	34,116	37.2	226	Integrase core domain protein (*Streptococcus parauberis*)	99 (226)	ZP_08245901
*orf31*	34,529	35,023	35.2	164	Hypothetical protein MobC (*Enterococcus faecalis*)	100 (164)	ZP_16756360
*orf32*	35,002	36,234	40.5	410	Relaxase/Mobilization nuclease domain/MobA (*Enterococcus* spp.)	99 (410)	CBL33209
*orf33*	36,231	36,854	37.2	207	Mobilization protein MobB (*Lactococcus lactis*)	94 (207)	YP_006998470
*orf34*	37,857	39,017	33.1	386	Replication protein RepB (pCIS2 *Lactococcus lactis*)	83 (386)	YP_006998536.1
*Δorf*	37,072	37,473	31.8	133	Mobilization/filimentation protein Fic (pCIS8 *Lactococcus lactis*) (200 aa)	100 (133)	YP_006998469
*orf35*	39,010	39,729	34.8	239	Plasmid replication-associated protein, RepX-like protein (pAF14 *Lactococcus lactis*)	50 (239)	YP_006964748
*orf36*	39,740	358	38.0	1025	Type I restriction-modification system, deoxyribonuclease subunit/HsdR (*Lactococcus lactis*)	99 (1025)	AAB91415

^a^Including start and stop codons.

^b^The overall G+C content of pLG9 and pLG42 is 32.6% and 36.04%, respectively.

^c^aa, amino acids

**Fig 3:**
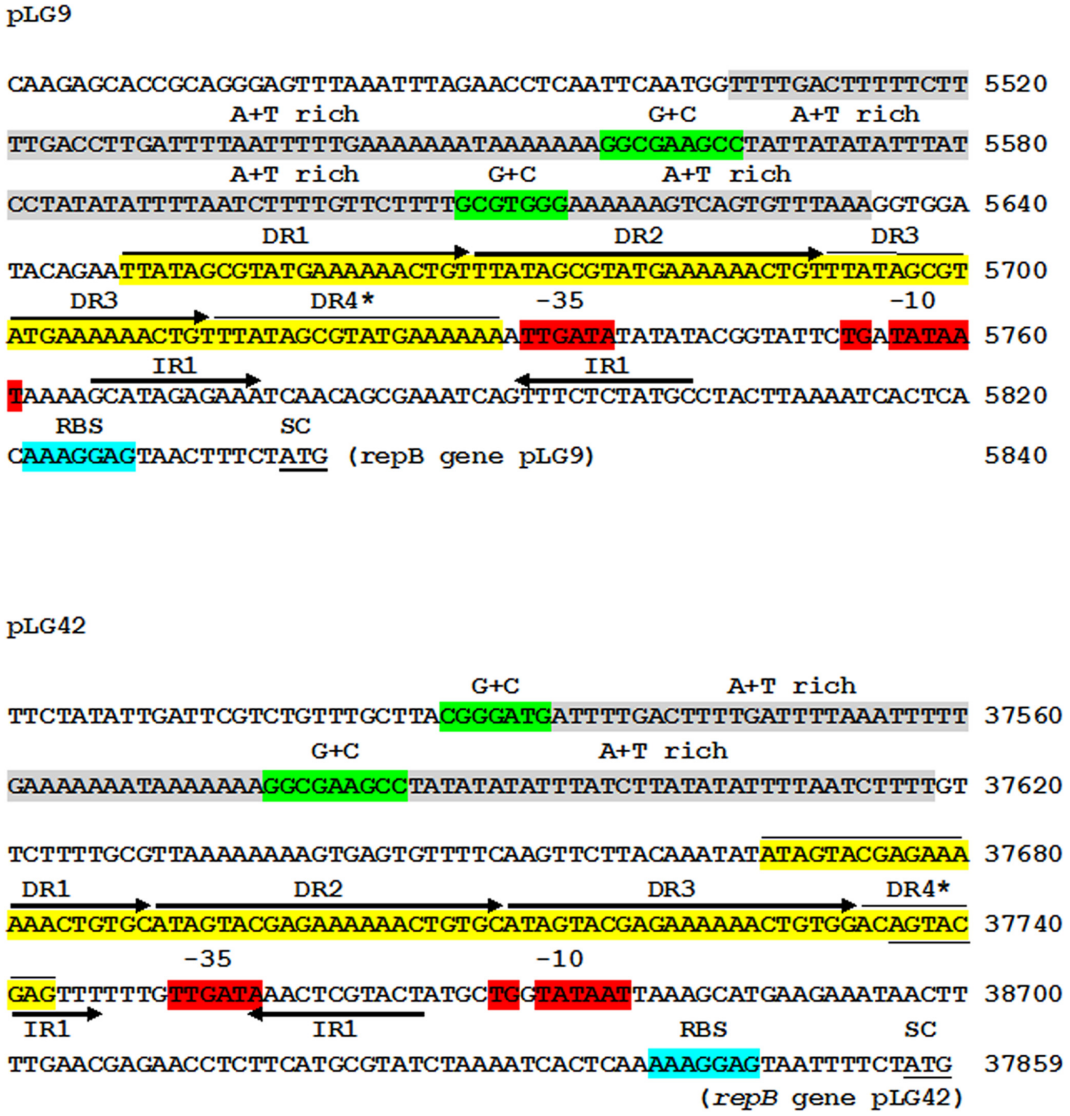
Detailed DNA sequence at the *ori* region of pLG9 and pLG42. Direct (DR) and inverted (IR) repeats are indicated by head to tail and head to head arrows, respectively. DR4* indicates incomplete repeats at the *ori* region of both pLG9 and pLG42. AT- and GC-rich sequences are colored in gray and green, respectively. Putative promoter and ribosome binding sites (RBS) sequences are colored in red and purple, respectively. Start codons of each of the *repB* genes appears in bold and underlined.

**Fig 4:**
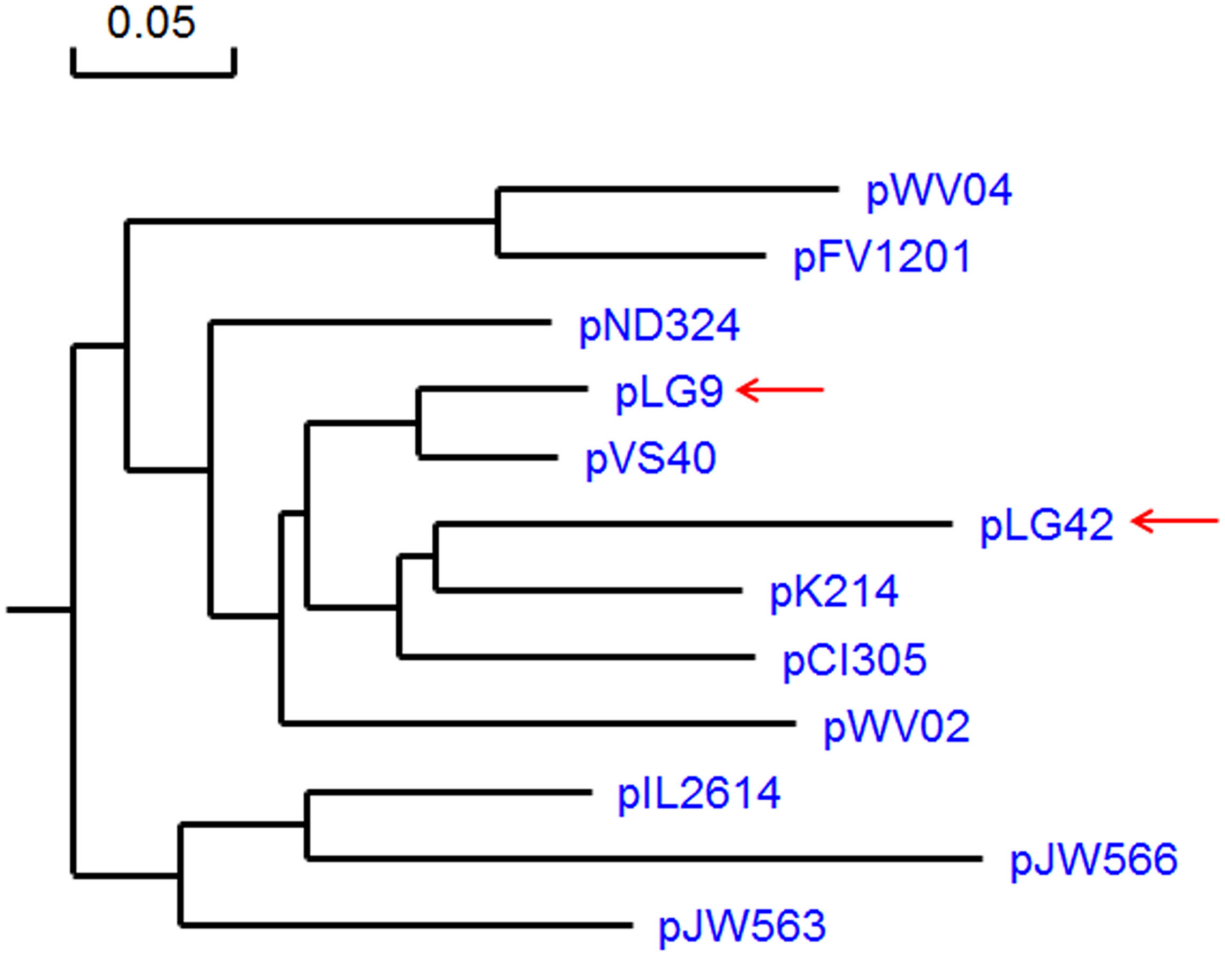
Phylogenetic relationships of Rep proteins from pLG9 and pLG42 of *L*. *garvieae* 31405 (marked by arrows) and prototype plasmids from *Lactococcus lactis* strains. The rooted phylogenetic tree was calculated by the sequence distance method using the neighbor-joining algorithm and a bootstrapping trial number of 1000.

Based on the homology and structural organization of translated and untranslated sequences at their putative *ori* region, pLG9 and pLG42 are predicted to replicate via a theta replication mechanism (Fig 3). Upstream of each respective *repB* gene, large AT-rich regions containing GC-rich clusters were observed in both pLG9 and pLG42, followed by a typical 22 bp perfect direct repeat (DR) specific to each plasmid. These DRs (iterons) are repeated three and a half times in each of the plasmids, a standard characteristic of many *L*. *lactis* plasmids [30–32]. Downstream of these sequences, well conserved promoter and ribosome binding site (RBS) existed (Fig 3). The plasmids’ deduced Rep proteins belonged to the Rep_3 superfamily (pfam01051) and the RepB_C family (pfam06430) respectfully. Multiple alignments of RepB sequences from pLG9 and pLG42 with those from well-recognized theta-type replicons of *L*. *lactis* [30] indicated small phylogenetic distances to plasmids of this species. In terms of the replication proteins, the closest relatives to pLG9 and pLG42 among prototype plasmids of *L*. *lactis* were pVS40 and pK214, respectively (Fig 4). However, all plasmids in this analysis showed an amino acid identity of their Rep proteins of >77.5%. Downstream of *repB* in lactococcal theta-replicating plasmids there is often a conserved gene referred to as *orfX* [30, 32]. *ofrX* is linked to *repB* by a small overlap, and its product has an N-terminal helix-turn-helix motif, characteristic of DNA binding proteins. This gene is dispensable but could affect the plasmid copy number, plasmid stability or both [30].

No set of genes encoding the required conjugation machinery for self-transmission could be identified in either of the plasmids. However, pLG42 harbours a complete set of genes for mobilization: *orf*s 31 to 33. These encode the MobC, MobA and MobB proteins respectively. The genes appear to be transcriptionally coupled since the stop codon of the preceding gene overlaps the start codon of the following one. Immediately upstream of *mobC*, a region of around 170 bp (nt position 34350–34520) was found containing three inverted repeats (IR) plus a DR identical to those of the *oriT* region of pNZ4000 (AF03685). This region includes the postulated *nic* site (hexamer CTTGCA) just downstream of a conserved pair of IRs. The functionality of the *oriT* sequence in pNZ4000 has been experimentally demonstrated [33]. A 5’ interrupted version of an identical *oriT* sequence is also present immediately upstream of a truncated *mobC* gene at nt position 4712 in pLG9.

A conjugative transposon encoding tetracycline resistance by a *tet*(S) gene had been observed in the genome sequence of *L*. *garvieae* IPLA 31405 [20]. Mating experiments were performed in order to check whether pLG42 could be mobilized by the transposon machinery, which might act as a sexual factor [34], from IPLA 31405 to *L*. *lactis* subsp. *cremoris* MG1614. Lactose positive transconjugants were never obtained. Furthermore, efforts to transfer the tetracycline resistance from IPLA 31405 to *L*. *lactis* MG1614 and *Enterococcus faecalis* OG1RF proved to be unsuccessful (data not shown). Under the same experimental conditions, transference of the lactose plasmid from *L*. *lactis* subsp. *cremoris* NCDO712 to MG1614 occurred at a rate of 2.6 x 10^–6^ transconjugants per donor (not shown).

In contrast to the plasmid complement of the human pathogen *L*. *garvieae* 21881, which harbours several genes encoding proteins that could be considered putative virulence factors [18], neither pLG9 nor pLG42 showed any evidence of virulence-related genes.

### Dairy related phenotypes

Plasmids of *L*. *garvieae* have received far less attention than those of *L*. *lactis* [17, 18, 35] and thus little is known about them, what they encode, or about their biological significance. The traits that encourage proliferation in the dairy environment are well established for *L*. *lactis*; these play a central role in the use of this species as an industrial starter in dairy fermentations. The majority of these properties, including the ability to utilize lactose and casein, and resistance against bacteriophage attack, are reported associated with plasmids (for a review see [9]).

Genes encoding β-galactosidases or related enzymes that can split lactose into its constituent monomers have yet to be reported in *L*. *garvieae* genomes [36], and none were found in the pLG9 and pLG42 host strain [20]. However, a full set of genes predicted to code for the lactose-specific phosphoenolpyruvate-phosphotransferase system (PEP-PTS), contained within the operon *lacABCDFEGX*, as well as the putative divergently transcribed repressor-encoding gene *lacR*, were located in pLG42. After incorporation to the cell, lactose is hydrolyzed in this system by a phospho-β-galactosidase (encoded by *lacG*). The lactose cluster was flanked by intact copies of two IS elements, IS*240* and IS*1216* (Fig 2), which suggested it was horizontally transferred as a block. Lactose utilization [37, 38] and hybridisation and PCR experiments using lactose-encoding genes as probes or targets [39, 40] have shown the ability to metabolise lactose, and indeed the lactose operon, to be widely distributed among *L*. *garvieae* dairy strains (although it is not exclusive to such strains [36]). Indeed, hybridisation experiments in this work using a segment of the phospho-β-galactosidase gene as a probe recorded a positive hybridisation signal in plasmids of different sizes in lactose-positive dairy isolates *L*. *garvieae* N201 and 1042 (data not shown). However, no signal was found for the lactose-negative strains *L*. *garvieae* 1204 and DK2-25. In contrast to the results of this study, the *lacG* gene has been reported to be chromosomally encoded on dairy strains isolated from Italian cheeses [39]. Lactose is the only abundant carbohydrate in milk; the ability to utilise lactose would therefore provide dairy *L*. *garvieae* with a key physiological advantage in terms of adapting to the dairy environment. An HGT event may have allowed *L*. *garvieae* to acquire the lactose-utilizing phenotype [40]. Sequence analysis of the lactose module in pLG42 and its nucleotide similarity to several *L*. *lactis* plasmids, reinforces the idea that this latter species was involved in an HGT episode.

Phage infection of technologically important LAB is the main cause of acidification failure in the manufacture of fermented dairy products such as butter, buttermilk, cheese, and yoghurt [41]. The prevalence of phages able to attack species of LAB in raw and pasteurised milk samples is very high [42]. Phage-resistant *L*. *lactis* strains therefore continue to be pivotal in the search for new LAB starter candidates. Not surprisingly, given the possible spread of resistant phenotypes among starter LAB, phage resistance mechanisms, and particularly those of *L*. *lactis*, have received great attention [41]. Restriction/modification (R/M) systems appear to be common phage-resistance mechanisms in *L*. *lactis*, and their genetic determinants are frequently carried on plasmids [43]. Our knowledge of the types and loads of phages infecting *L*. *garvieae* is scant [44, 45], but they might be expected to be abundant in liquid ecosystems such as water and milk. Therefore, an R/M system might also help protect *L*. *garvieae* against phage infection. An operon encoding a complete Type I R/M system was found in pLG42 (Fig 2). This system consists of three proteins: HsdR (a restriction subunit), HsdM (a methylation subunit) and HsdS (a specificity subunit). The holoenzyme of Type I R/M systems consists of a heterooligomer of HsdM and HsdR subunits; this can be joined by different HsdS subunits to broaden its specificity and phage resistance [28]. A gene coding for a distinct HsdS subunit is present in pLG9. Further, at least part of another Type I R/M system consisting of two HsdR subunits and an HdsM subunit is encoded within the *L*. *garvieae* IPLA 31405 chromosome (y*7c_102275*, *y7c_103985*, and *y7c_118143*) (AKFO00000000.1). Additionally, nucleases of the GIY-YIG superfamily (*orf8* in pLG9) can function like those of R/M systems by cleaving foreign DNA at specific target sites [46]; this might also enhance resistance against phage infection. However, no evidence of any methyltransferase, which would render the *L*. *garvieae* DNA resistant to the GIY-YIG nuclease, was identified. Alternatively, the GIY-YIG endonuclease might be a remnant of a homing-like, site-specific mobile genetic element split between pLG9 (endonuclease) and pLG42 (the *Δorf* ion-binding domain).

Several genes encoding ATPases and other components of efflux system, which might be involved in resistance to metal ions, such as cobalt, lead, cadmium, nickel and mercury, were identified in the sequence of pLG42. *orf3* encoded a CorA-like protein (pfam01544) with homology to proteins implicated in the transport of divalent cations such Mg^2+^ and Co^2+^. In addition, *orf25* to *orf29* might encode a MerR-like, P-type ATPase system with the potential to transport copper, lead, cadmium, and/or zinc. Ion transporters might primarily participate in maintaining cation homeostasis by allowing their regulated efflux from the cell [13]. Alternatively, some of these metals might act as cofactors in enzymes, and transporters might enhance survival of host cells by facilitating uptake of cations in metal-depleted environments [47].

### Plasmid copy number

The relative copy number of pLG9 and pLG42 was determined in exponentially growing cells and cultures at the stationary growth phase by qPCR targeting the genes encoding their respective replication proteins. A 10-fold serial dilution of total DNA isolated from *L*. *garvieae* IPLA 31405 was used to construct standard curves for GADPH, EF-Tu, *repB*-pLG9 and *repB*-pLG42 genes. Theoretically, for a 10-fold dilution of template DNA, a cycle threshold (*Ct*) value of 3.32 would be expected [25]. The amplification efficiency of control and target genes was linear (R^2^>0.99) in the range tested. Additionally, the *Ct* values obtained resulted in small differences from the theoretical values (98.5%, 98.0%, 97.2% and 99.6% for the GADPH, EF-Tu, *repB*-pLG9 and *repB*-pLG42 genes, respectively). The calculated copy numbers per chromosome equivalent of pLG9 and pLG42 were 2 and 5, respectively. This agrees rather well with the results of plasmid profile analysis for IPLA 31405 obtained using lysis and agarose gel electrophoresis, in which a weaker band for pLG9 was always observed (Fig 1).

### Plasmid stability and phenotype

The stability of the two plasmids in a rich medium was examined by PCR. After passing through around 100 generations in non-selective conditions (GM17), all the analysed colonies retained both pLG9 and pLG42. Therefore, even under laboratory conditions, both plasmids proved to be very stable. After several rounds, a single segregant losing pLG42 was obtained. Phenotypic analysis showed this derivative unable to grow in or acidify lactose-containing media, further evidencing the location of the PEP-PTS operon and phospho-β-galactosidase gene in pLG42. However, no differences were seen between the parental and the pLG42-free derivative in terms of their sugar fermentation patterns. Apart from lactose, IPLA 31405 and its pLG42-cured derivative were both positive for the fermentation of xylose, galactose, glucose, fructose, mannose, mannitol, N-acetyl-glucosamine, amygdalin, arbutin, esculin, salicin, cellobiose, maltose, sucrose, gentiobiose, D-tagatose, and gluconate. The genetic basis for the fermentation of these carbohydrates had already been associated to the IPLA 31405 genome [20]. Similarly, parental and pLG42-negative strains were found to share identical susceptibility/resistance profiles with respect to a series of heavy metal ions. MICs of >2048 μg/ml were obtained for all heavy metals tested in both strains, except for mercury (2 μg/ml) and cobalt (64 μg/ml). The latter results suggest that either the pLG42-encoded ion transporters are non-functional or that sufficient transport activity is provided by other genes encoded by the chromosome, where more than 18 *orf*s were found to encode proteins with similarity to metal transporters (ABC, ATPase, and other types) with different specificity [20].

**Fig 5:**
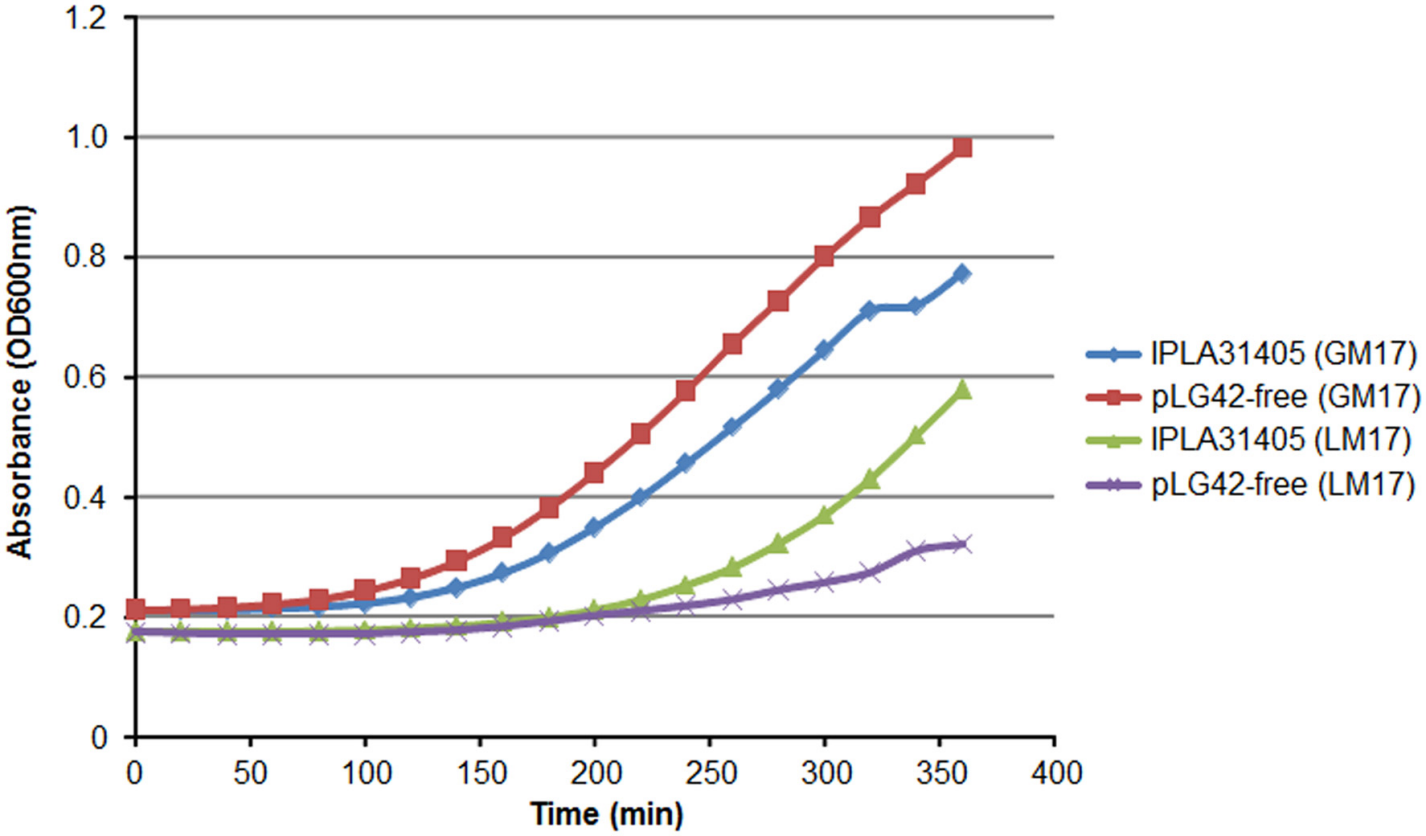
Growth of *L*. *garvieae* IPLA 31405 and its pLG42-free derivative in GM17 and LM17 at 30°C. The maximum growth rate (μ_max_) was calculated with the formula (ln x_1_—ln x_0_)/(t_1_—t_0_), where x_0_ and x_1_ are the optical density at 600 nm at t_0_ and t_1_, respectively.

Growth of parental and pLG42-free strains in GM17 broth proved they reached approximately the same final optical density after 24 h (OD_600_ of around 3.0). However, the pLG42-free derivative showed a slightly higher maximum growth rate during the logarithmic phage (0.373 as compared to 0.370 for the parental strain) (Fig 5). Large lactococcal plasmids are thought to be a metabolic burden for the host cell [48]. Thus, it is assumed that plasmid maintenance is based on adaptive evolutionary pressure in the dairy environment [10, 14]. On the contrary, in LM17, the parental strain grew faster and attained a higher cellular density after 24 h (OD_600_ 2.25 *versus* 0.69 for pLG42-free strain). In addition to lactose utilization, rapid and efficient growth in milk also requires the ability to degrade and metabolise casein via a cell wall-associated protease (PrtP) with caseinolytic activity and an oligopeptide transport system [49]. Although a number of genes encoding proteases and peptidases, plus components of an oligopeptide transport system similar to the Opp seen in *L*. *lactis*, were identified in the *L*. *garvieae* IPLA 31405 genome, genes encoding PrtP and its maturase protein (PrtM) were not detected [20]. This agrees well with the poor growth and scarce acidification activity in milk compared to the proteinase-positive *L*. *lactis* of *L*. *garvieae* strains, including IPLA 31405, which has already been reported [38]. Furthermore, addition of yeast extract to milk was shown to increase the acidification attained by IPLA 31405 (pH at 23 h of 4.95 with *versus* 6.75 without yeast extract) (S1 Fig). However, addition of yeast extract to milk had only a marginal influence in the final pH obtained by the pLG42-free derivative.

The lack of a collection of species-specific phages infecting *L*. *garvieae* makes difficult checking the involvement of pLG42-encoded systems in phage resistance. In spite of this, the parental and pLG42-free derivative were challenged against a set of 36 purified phages and phage lysates from industrial sources infecting starter strains of *L*. *lactis*. Surprisingly, both strains proved to be resistant against the same five phages and susceptible to 31 (though with high variability from phage to phage in the size of the inhibiting halo). Moreover, 11 halos of clearance were shown to be bigger for the original strain, suggesting the loss of pLG42 entails a certain degree of phage protection. The apparent greater resistance to phages of pLG42-free strain might be due to its higher maximum growth rate during the logarithmic phage. The strong susceptibility of *L*. *garvieae* to *L*. *lactis* phages and infective whey deserves further investigation. At present, the involvement of endolysin activity rather than the lytic phages themselves cannot be discarded.

## Conclusions

The plasmid complement-pLG9 and pLG42- of *L*. *garvieae* IPLA 31405 was examined at the molecular level and the results deposited in the GenBank database. Type of replication, stability and copy number make replicons of pLG9 and pLG42 excellent candidates for the development of cloning vectors for *L*. *garvieae*. Both plasmids, but particularly pLG42, evolved as a collection of functional gene cassettes. The gene modules are interspersed with an array of complete and truncated IS elements. The genetic determinants that allow *L*. *garvieae* to utilize lactose efficiently are of paramount importance to its growth and survival in the milk environment. However, involvement of plamidic R/M system in phage resistance was not demonstrated. Except for minor segments of pLG9, genes identified in both plasmids showed high homology to those in the genome of dairy niche LAB species (*Lactococcus* spp., *Enterococcus* spp., and *Streptococcus thermophilus*) (Table 2). This suggests the existence of a supragenome shared across dairy bacteria that allows them to better adapt to the competitive and stressful dairy ecosystems.

## Supporting Information

S1 Fig Acidification of milk by *Lactococcus garvieae* IPLA 31405 and its pLG42-free derivative with and without addition of yeast extract (0.5%). (TIF)
