# Supplementary figures and images for: The Plasmid Complement of the Cheese Isolate Lactococcus garvieae IPLA 31405 Revealed Adaptation to the Dairy Environment
Source: PLoS One. 2015 May 5;10(5):e0126101. doi: 10.1371/journal.pone.0126101 (PMC4420470; doi:10.1371/journal.pone.0126101)

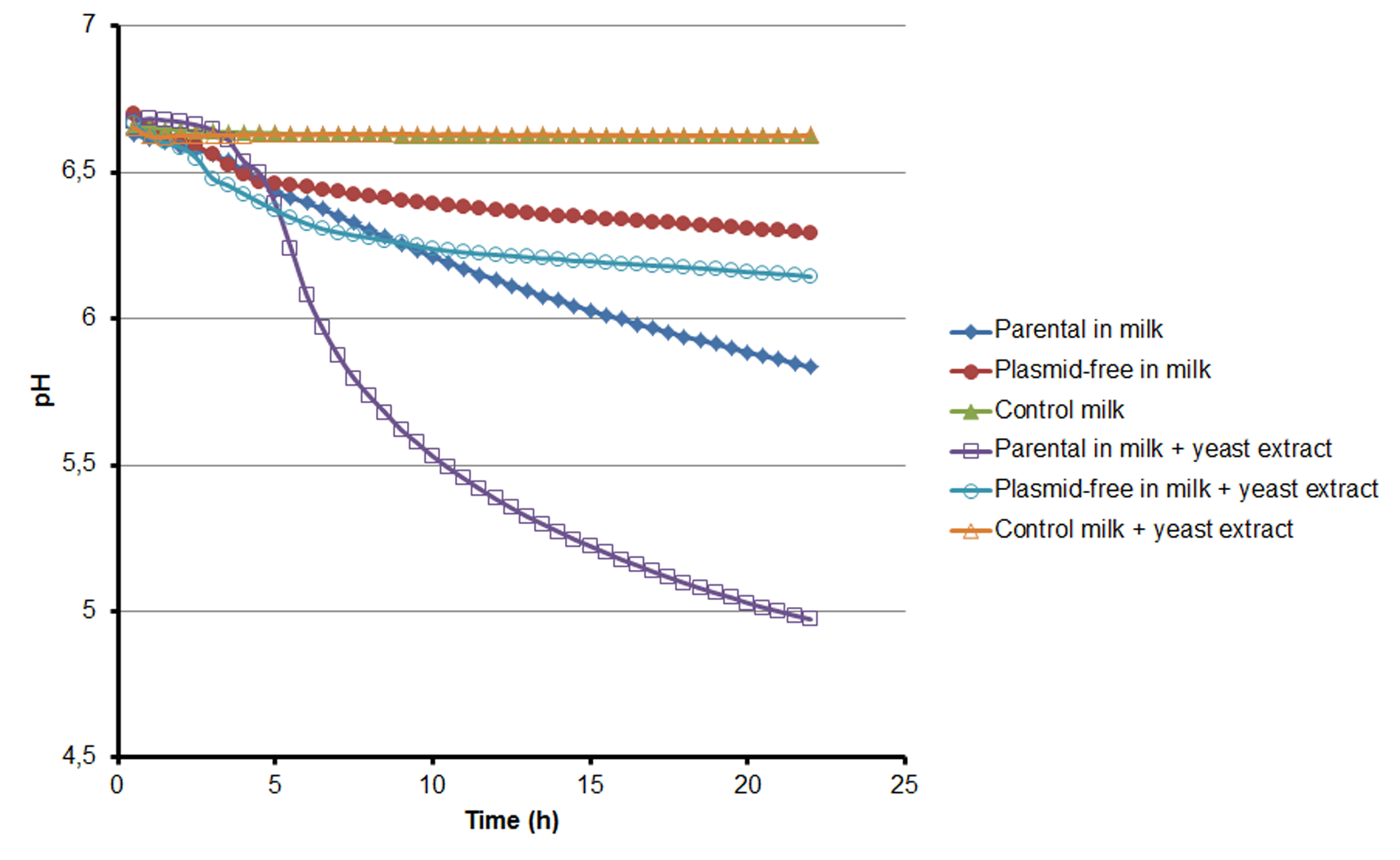

Supplement: S1 Fig — (TIF) [file pone.0126101.s001.tif]
